# The Reliability of the Apple Watch’s Electrocardiogram

**DOI:** 10.7759/cureus.49786

**Published:** 2023-12-01

**Authors:** Sara Alnasser, Dalal Alkalthem, Sara Alenazi, Muneera Alsowinea, Narin Alanazi, Ahmed Al Fagih

**Affiliations:** 1 Clinical Sciences, Princess Noura Bint Abdulrahman University, Riyadh, SAU; 2 Cardiology, Prince Sultan Military Medical City, Riyadh, SAU

**Keywords:** sensitivity, reliability, electrocardiogram, apple watch, smartwatch

## Abstract

Background

An electrocardiogram (ECG) is a standard tool used to detect various cardiovascular abnormalities. Detection sensitivity for atrial fibrillation (AF) was recently shown to be greatly increased by using short, intermittent ECG recordings.^ ^Modern mobile ECG recording devices that can monitor patients' heart activities around the clock have made this a reality. The Apple Watch is one of these portable ECG devices that can detect heart rhythms and is approved by the American FDA for screening and detecting AF.

Objectives

We compared the results of the Apple Watch I lead ECG with conventional ECG results to assess the sensitivity and specificity of the Apple Watch I lead ECG. We then determined the abnormalities that can be detected by the Apple Watch I lead ECG.

Methods

This study was conducted on outpatient cardiac clinics at King Abdullah bin Abdulaziz University Hospital (KAAUH), and Prince Sultan Cardiac Center (PSCC), from May to October 2021. A standard 12-lead ECG was recorded and compared with the Apple Watch I lead ECG. A total of 469 ECG comparisons were included in this study and evaluated by two investigators. The data on patient demographics, medical and medication history, and ECG data were reviewed and analyzed using IBM SPSS Statistics for Windows, Version 23 (Released 2015; IBM Corp., Armonk, New York, United States).

Results

No significant differences were seen between the Apple Watch and the 12-lead ECG in terms of the studied ECG characteristics. A significant and strong positive correlation between the heart rate measurements in the 12-lead ECG and Apple Watch ECG was documented. The most commonly found abnormalities in the Apple Watch ECG were AF in 37 (7.9%), followed by first-degree atrioventricular (AV) block in 32 (6.8%). The sensitivity of Apple Watch's automated interpretation to detect an AF was 99.54%, while the manual interpretation yielded a sensitivity of 100%.

Conclusion

The results of this study demonstrated a robust relationship between the 12-lead ECG and Apple Watch ECG in the diagnosis of arrhythmias. Consequently, cardiac patients may consider the Apple Watch ECG a trustworthy remote monitoring technique.

## Introduction

Today's physicians typically employ a 12-lead electrocardiogram (ECG) to diagnose heart problems. However, it is possible that issues with the heart would not show up on a conventional 10-second recording [1]. Recent research has shown that atrial fibrillation (AF) can be detected with far higher sensitivity using multiple brief ECG recordings spread out over a longer period of time as opposed to only one long recording [2]. Now that transportable ECG recording devices exist, doctors may constantly monitor their patients' heart rates and upload the data to a cloud service for analysis [1].

The Apple Watch is one such portable ECG device that can detect heart rhythm and is approved by the American FDA for screening and detecting AF. It has two electrodes; one is impeded in the back of the watch in continuous contact with the wrist, and the other one is in the digital crown. The circle is closed by holding the right index finger on the digital crown for 30 seconds. The results are represented in the application as an ECG waveform that corresponds to lead I [3]. 

Innovation and advancement of new technologies in this generation have made smartwatches an integral part of daily life. In the United States, 13% of people have smartwatches, and an additional 40% express interest in purchasing one [4]. There are expected to be 46 million smartwatch owners in the Middle East and Africa by 2022 [5]. The Apple Watch is the most popular smartwatch in the market [6]. Since cardiovascular disease is one of the leading causes of death globally [7], it stands to reason that new technology will make it possible to screen for diseases in an efficient manner, thereby preventing illness and saving lives. 

Previous studies assessed the ability of the Apple Watch to identify AF and found that it has a sensitivity of 98% versus standard 12-lead ECG [3]. The positive predictive value (PPV) of Apple Watch notifications was 84% [8]. The fact that the ECG on the smartwatch was of such great quality was also fascinating. Specifically, no substantial baseline artifacts were present in any of the smartwatch's ECG tracings [9]. The existence or lack of ischemia needs a 12-lead ECG to be diagnosed and numerous-lead wearable devices are expected to be superior to single-lead devices. However, it still has a chance to interpret ischemia using portable one or three-lead ECGs with some degree of uncertainty in comparison to a 12-lead ECG [10]. Furthermore, it has been reported that Apple Watch identified an ST-depression in a patient with typical angina symptoms with negative initial 12-lead ECG and troponin [11]. Moreover, there has been an interesting case report of a patient with symptomatic ST elevation myocardial infarction (STEMI) who did not consider her symptoms to be of cardiac origin, but her Apple Watch alert caused her to seek medical care [12]. 

The Apple Watch can also identify a re-entrant supra-ventricular tachycardia during a symptomatic event that was not identified by outpatient rhythm monitoring [13]. It has also been reported, although not authorized by the US FDA, that the Apple Watch can be used to test for and monitor QT prolongation [14,15].

The Apple Watch can acquire an ECG in one lead or three leads, and there was a high degree of agreement between the smartwatch ECG and the gold standard ECG for every segment in all three leads that were analyzed. When regular ECG facilities are not immediately available, this method can be advised as a new technique for speedier diagnosis of cardiac diseases that may be identified from leads I-III ECG [9,16].

The trend toward tele/remote health continues especially after the COVID-19 pandemic and quarantine regulations. Such a strategy can enhance the delivery of at-home healthcare and can combine telehealth with portable ECG technologies [17].

## Materials and methods

Data collection

This study was conducted on outpatient cardiac clinics at King Abdullah bin Abdulaziz University Hospital (KAAUH), and Prince Sultan Cardiac Center (PSCC), from May to October 2021. Information about patients' ages, sexes, sizes, medical histories, medications, and conditions was gathered from the outset. Disease history was taken by asking the participants if they were known to have or were diagnosed with diabetes mellitus, hypertension, heart disease, or dyslipidemia. Heart diseases that can be diagnosed by ECG include arrhythmia, coronary artery disease, heart attack, cardiomyopathy, and other diseases. As for medication history, it was taken from a chart review of patients' medical records.

ECG recording

The common ECG device used to record a typical 12-lead ECG. After that, a lead I ECG was taken using a Series 6 Apple Watch. The participant’s left wrist hosted the watch while his right index finger rested on the crown. After 30 seconds of recording, the participant's watch was taken off. All ECGs were captured using an iPhone's Health app and then forwarded as a PDF file containing participant information. The data was utilized for comparing the heart rate information from the watch to a reference ECG. Calculations of sensitivity and specificity were made at the conclusion.

ECG interpretation

The PR interval is the time between the beginning of the P-wave and the beginning of the QRS complex in an ECG. This represents how long it takes for the heart to receive excitement from the sinoatrial (SA) node and send it on to the ventricle. The PR interval typically lasts between 0.1 and 0.2 seconds (three to five tiny squares). More than 0.2 seconds (five tiny squares) is considered a protracted PR interval. Excitation in the ventricle takes time, which is reflected in the length of the QRS complex. QRS complexes often last less than 0.12 seconds. More than 0.12 seconds (3 pts) is considered a wide QRS complex. If conduction is sluggish, this will happen. The interval following the QRS complex and prior to the onset of the T-wave is referred to as the ST segment. More than 1 mm above the isoelectric line is considered ST elevation, and more than 0.5 mm below the isoelectric line is considered ST depression. The research team standardized these definitions and received instruction in ECG interpretation. Two researchers will look through each ECG article and come to a consensus on how it should be interpreted. Questions about ECG interpretation were sent to a cardiac consultant. 

Statistical analysis

IBM SPSS Statistics for Windows, Version 23 (Released 2015; IBM Corp., Armonk, New York, United States) was used for the data analysis. Categorical variables were represented by frequencies and percentages. Statistical data was displayed using measures of central tendency and dispersion, respectively. To determine whether or not there were statistically considerable differences in the overall distribution of results between categories, a test was performed known as Chi-square. Correlations between quantitative variables were also analyzed using Pearson's correlation. A significance level of 0.05 was chosen. 

## Results

Sociodemographic profile of the participants

There were 469 participants in the study. Table 1 shows the sociodemographic profile of the participants. The mean age of participants was 56.43 + 16.3 years; 264 (56.3%) were males, and 205 (43.7%) were females. BMI data showed that 104 (22.2%) had a normal weight, 139 (29.6%) were overweight, 204 (43.5%) were obese, and 22 (4.7%) did not have a documented BMI. There were 298 (63.5%) participants with one or more heart diseases, 131 (27.93%) with coronary artery disease, 57 (12.2%) with arrhythmia, 53 (11.3%) with cardiomyopathy, and 84 (17.9%) with other cardiac diseases. 

**Table 1:** Sociodemographic profile of the participants (n = 469).

Sociodemographic profile of the participants (n = 469)		
Demographic Characteristics	n	%
Age		
Mean	56.43	
Standard deviation	16.30	
Gender		
Male	264	56.30
Female	205	43.70
BMI		
Normal weight	104	22.20
Overweight	139	29.60
Obesity	204	43.50
Undocumented	22	4.70
Co-morbidities		
Heart Disease	298	63.5
Coronary Artery Disease	131	27.93
Arrhythmia	57	12.2
Cardiomyopathy	53	11.3
Others	84	17.9
Dyslipidemia	255	54.4
Hypertension	240	51.2
Diabetes Mellitus	209	44.6
Thyroid Disease	71	15.1
Asthma	65	13.9
Other Disease	105	22.4
Use of Drugs		
Use of any drug	411	87.6
Angiotensin receptor blockers (ARBs) and/or angiotensin-converting enzyme inhibitors (ACEI)	206	43.9
Anti-arrhythmic drugs (including beta blocker and calcium-channel blockers)	328	69.9

There were 255 participants (54.4%) with dyslipidemia, 240 (51.2%) with hypertension, 209 (44.6%) with diabetes mellitus, 71 (15.1%) with thyroid disease, 65 (13.9%) with asthma, and 105 (22.4%) had other diseases. Here, 411 (87.6%) of the participants reported using a drug: The most commonly reported drugs were anti-arrhythmic drugs for 328 (69.9%) including beta blockers and calcium channel blockers while 206 (43.9%) were using angiotensin receptor blockers (ARBs) and/or angiotensin-converting enzyme inhibitors (ACEIs).

ECG finding comparison between the 12-lead ECG and Apple Watch ECG

Table 2 compares ECG findings between a 12-point lead ECG and Apple Watch ECG. Data on the rhythm showed that of the 12-point lead ECG, seven (1.5%) had regularly irregular rhythm. In the Apple Watch, four (0.9%) had regularly irregular rhythm. Moreover, in the 12-lead ECG, 44 (9.4%) had irregularly irregular rhythm, while in the Apple Watch 49 (10.4%) had irregularly irregular rhythm. The P-wave was absent in 39 (8.3%) in the 12-lead ECG. It was absent in 52 (11.1%) in the Apple Watch ECG. As for the P-R interval, it was constant in 389 (82.9%) in the 12-lead ECG, but it was constant in 381 in the Apple Watch ECG (81.2%). As for the QRS complex width, in the 12-lead ECG, it was wide in 16 (3.4%), but it was wide in 24 (5.1%) in the Apple Watch. No significant differences were seen between the Apple Watch and the 12-lead ECG with regard to rhythm, presence or absence of P-wave, P-R interval, and QRS complex width. The mean heart rate in the 12-lead ECG was 72.67 + 13.02 bpm, while the mean heart rate in the Apple Watch ECG was 74.14 + 12.63 bpm. Pearson’s correlation showed a significant and highly strong positive correlation (agreement) between the heart rate measurements in the 12-lead ECG and the Apple Watch ECG. 

**Table 2:** ECG finding comparison between the 12-lead ECG and Apple Watch ECG (n = 469).

ECG finding comparison between the 12-lead ECG and Apple Watch ECG (n = 469)														
		12-lead ECG				Apple Watch ECG				p-value				
Item		n		%		n		%						
Rhythm														
Regular	418		89.10		416		88.70		0.579					
Regularly irregular	7		1.50		4		0.90							
Irregularly irregular	44		9.40		49		10.40							
P-wave														
Present	430		91.70		417		88.90		0.152					
Absent	39		8.30		52		11.10							
P-R interval														
Constant	389		82.90		381		81.20		0.826					
Fixed prolongation	35		7.50		32		6.80							
Progressive prolongation	2		0.40		2		0.40							
NA	41		8.70		51		10.90							
Variable change	2		0.40		3		0.60							
QRS complex width														
Normal	453		96.60		445		94.90		0.196					
Wide	16		3.40		24		5.10							
Heart Rate														
Mean	72.67				74.14									
Standard deviation	13.02				12.63									
P-value	<0.001*													
Correlation coefficient	0.903													

Table 3 shows the ECG impression comparison between the 12-lead ECG and Apple Watch ECG. In the 12-lead ECG, 342 (72.9%) had a normal ECG, while 334 (71.2%) had a normal ECG in the Apple Watch ECG. The most commonly found abnormalities in the 12-lead ECG were: first-degree AV block in 35 (7.5%) followed by AF in 34 (7.2%), premature ventricular contraction in 20 (4.3%), and wide QRS complex in 13 (2.8%). The most commonly found abnormalities in the Apple Watch ECG were AF in 37 (7.9%) followed by first-degree AV block in 32 (6.8%), premature ventricular contraction in 21 (4.5%), and wide QRS complex in 17 (3.6%).

**Table 3:** ECG impression comparison between the 12-lead ECG and Apple Watch ECG (n = 469). AV: Atrioventricular

ECG impression comparison between the 12-lead ECG and Apple Watch ECG (n = 469)						
		12-lead ECG		Apple Watch ECG		
Impression		n	%	n	%	
Normal ECG		342	72.90	334	71.20	
First-degree AV block		35	7.50	32	6.80	
Atrial fibrillation		34	7.20	37	7.90	
Premature ventricular contraction		20	4.30	21	4.50	
Wide QRS complex		13	2.80	17	3.60	
Sinus arrhythmia		9	1.90	12	2.60	
Atrial flutter		6	1.30	2	0.40	
Third-degree AV block		2	0.40	1	0.20	
Sinus tachycardia		2	0.40	3	0.60	
Atrial tachycardia		2	0.40	0	0.00	
Paced rhythm		2	0.40	1	0.20	
Regular no P-wave		4	0.80	12	2.50	
Premature atrial complex		1	0.20	2	0.40	
Sinus node dysfunction		1	0.20	1	0.20	
Trigeminy		1	0.20	0	0.00	
Artifacts		0	0.00	5		

Sensitivity and specificity of the Apple Watch ECG to detect ECG abnormalities

Table 4 displays the sensitivity and specificity of Apple Watch ECG to detect ECG abnormalities. The sensitivity of Apple Watch ECG toward detecting an abnormal ECG was 88.19%, and the specificity was 92.98%. The sensitivity of Apple Watch ECG toward detecting first-degree AV block was 80%, and the specificity was 99.08%. The sensitivity of Apple Watch ECG toward detecting AF was 100% with a specificity of 99.18%. The sensitivity of Apple Watch ECG toward detecting premature ventricular contraction was 55%, and the specificity was 97.77%. The sensitivity of Apple Watch ECG toward detecting a wide QRS complex was 76.92%, and the specificity was 98.68%.

**Table 4:** Sensitivity and specificity of the Apple Watch ECG to detect ECG abnormalities (n = 469). AV: Atrioventricular

Sensitivity and specificity of the Apple Watch ECG to detect ECG abnormalities (n = 469)				
	Sensitivity and Specificity of the Apple Watch ECG Toward Detecting an Abnormal ECG			
	True Positive		112	
	False Positive		24	
	True Negative		318	
	False Negative		15	
	Sensitivity		88.19%	
	Specificity		92.98%	
	Sensitivity and specificity of Apple Watch ECG toward detecting first-degree AV block			
	True Positive		28	
	False Positive		4	
	True Negative		430	
	False Negative		7	
	Sensitivity		80%	
	Specificity		99.08%	
	Sensitivity and specificity of Apple Watch ECG toward detecting atrial fibrillation			
	True Positive		101	
	False Positive		3	
	True Negative		365	
	False Negative		0	
	Sensitivity		100%	
	Specificity		99.18%	
	Sensitivity and specificity of Apple Watch ECG toward detecting premature ventricular contraction			
	True Positive		11	
	False Positive		10	
	True Negative		439	
	False Negative		9	
	Sensitivity		55.00%	
	Specificity		97.77%	
	Sensitivity and specificity of Apple Watch ECG toward detecting wide QRS complex			
	True Positive		10	
	False Positive		6	
	True Negative		450	
	False Negative		3	
	Sensitivity		76.92%	
	Specificity		98.68%	

Sensitivity and specificity of Apple Watch interpretation to detect AF 

Within the context of screening test, it is important to avoid misconceptions about sensitivity, specificity, and predictive values. Table 5 shows the sensitivity and specificity of Apple Watch interpretation toward detecting an abnormal ECG. With a total of 469 Apple Watch ECG values, it came with a result of true positive (30), false positive (2), true negative (430), and false negative (7). The sensitivity of Apple Watch toward detecting an abnormal ECG was 81.08%, while the specificity was 99.54%.  

**Table 5:** Sensitivity and specificity of Apple Watch interpretation to detect atrial fibrillation (n = 469).

Sensitivity and specificity of Apple Watch interpretation to detect atrial fibrillation (n = 469)			
Sensitivity and specificity of Apple Watch interpretation toward detecting atrial fibrillation			
True Positive		30	
False Positive		2	
True Negative		430	
False Negative		7	
Sensitivity		81.08	
Specificity		99.54	

## Discussion

Innovations in sensor technologies have made it possible to record electric impulses from the heart in the absence of conventional ECG machines. Many such technologies are wearable and can record cardiac impulses for extended periods of time. These advancements can potentially enhance the utility of this technique in out-of-hospital settings [10].  

This study aimed to evaluate the validity of the Apple Watch ECG versus a 12-lead ECG. Our results showed that there were no significant differences in ECG readings between 12-lead ECG and Apple Watch ECG in terms of recording rhythm, P-wave, PR interval, and QRS width. Furthermore, there was a remarkable correlation between Apple Watch ECG and 12-lead ECG heart rate measurements.  

This study only included patients from a cardiac outpatient department in an attempt to detect more ECG abnormalities in Apple Watch other than AF, which in turn, will give the watch a more valuable role in remote monitoring of cardiac patients. However, other studies have included only healthy individuals [8,18-22]. Furthermore, these prior studies compared quantitative ECG characteristics such as duration and amplitude [9,21,22]. Here, we qualitatively compared ECGs by visually interpreting ECGs as a clinical interpretation and then comparing them.       

Participants in our study were mostly older (56 +16 years) and with heart disease (63.5%), some of them even had multiple cardiac comorbidities. This could be because data was collected from the cardiology outpatient department where patients are expected to be older and with other comorbidities. Furthermore, these patients represent a population who are more likely to benefit from such technologies in remotely monitoring their health. The Apple Heart Study is, to the best of our knowledge, the largest study that studied Apple Watch. It included a relatively healthier and younger population in which only 5.9% of participants were aged 65 or older while the majority of them (52%) were middle-aged between 22-39 years old. However, more than one-third of participants who received an irregular pulse notification were 65 years of age and older [4,8]. 

Our findings showed no significant difference between Apple Watch ECG and 12-lead ECG in any of the studied ECG characteristics: rate, rhythm, P-wave, PR-interval, and QRS width. Those characters were selectively studied because they can be relied on for diagnosis using one-lead ECG. The ST segment is another important character, but it is not a diagnostic finding from a one-lead ECG. However, several studies used the Apple Watch to record more than one lead ECG and detect ST changes with promising results [16,23]. Furthermore, there was more than one case report where an Apple Watch one-lead ECG recorded changes indicating cardiac ischemia or infarction [11,12]. 

The QT interval was previously studied and compared using Apple Watch and 12 lead ECGs [14,15]. The results showed agreement among the QT intervals of I, II, and V2 leads and the QT mean using the smartwatch and the standard ECG [15]. The other study used lead V6 instead of V2 and reported that QT measurements were adequate when the smartwatch was worn on the left wrist in 85% of patients; this figure increased to 94% when the smartwatch was moved to alternate positions [14]. 

There was a 10% difference between Apple Watch and 12-lead interpretations for P-wave. Although this difference was not significant, it is concordant with a previous study, which showed that the best correlation for the P-wave amplitude and visibility between the standard-ECG and the Apple-ECG is in lead V2. Therefore, the focus should be on lead V2 for interpreting the P-wave especially in cardiac arrhythmias [22]. Hence, future studies may compare Apple Watch ECG P-wave in lead I and V2. Although there were only five non-interpreted Apple Watch ECGs due to artifacts, some ECGs interpreted as sinus rhythm had many artifacts but on further review, there was a clear P-wave preceding QRS with regular rhythm indicating sinus rhythm.   

As a promising finding, we found that Apple Watch ECG can detect abnormalities other than AF based on our ECG characteristics. The most common abnormalities included first-degree AV block, premature ventricular contraction, and wide QRS complex. Thus, the decision to calculate sensitivity and specificity was affected by the frequency of these abnormalities. Specificity was always higher and better in first-degree AV block, premature ventricular contraction, and wide QRS complexes. However, AF had the highest sensitivity among others.  

The Apple Watch is commercially available to all populations, and thus we believe that high specificity is more important than sensitivity in such devices because an increase in false positives will increase the burden on healthcare facilities and will also increase the patient stress about these findings.  

There are limited studies evaluating these abnormalities. However, one study showed that a smartwatch can also help detect these abnormalities. This study showed a first-degree AV block in two Apple Watch tracings, and a wide QRS in one trace. This finding was confirmed to be a left bundle branch block on the corresponding 12-lead ECG. Furthermore, two participants had isolated premature atrial or ventricular contractions noted on AW or 12-lead ECG [21].  

Manual interpretation of the Apple Watch ECG for AF yielded a sensitivity of 100%. However, the automated interpretation yielded a sensitivity of 81%, which is even lower than the reported sensitivity in the FDA approval report (98.3%) [3]. This difference might be due to manual interpretation of the Apple Watch ECG by physicians, which would be more valuable.  

This study had some limitations to be highlighted. First, the risk of information bias as participants' medical history was taken verbally from them. However, the focus of this study was the ECG characteristic comparison. Another limitation is that ECG interpretation was done by junior non-cardiologist physicians. We recommend further and larger studies, overcoming these limitations to be done to build strong evidence on the reliability of such devices. 

## Conclusions

In this study, researchers found that the Apple Watch ECG was highly correlated with 12-lead ECG for the identification of arrhythmia. Additionally, 100% sensitivity was achieved through hand reading the Apple Watch ECG for AF, whereas 81% sensitivity was achieved through automatic interpretation. Thus, the Apple Watch ECG could be viewed as a trustworthy remote monitoring device for cardiac patients, particularly in situations where rapid access to regular ECG facilities is unavailable.
